# A Burmese Practice of Medicine

**Published:** 1910-08-06

**Authors:** 


### SPECIAL ARTICLES.

### A BURMESE PRACTICE OF MEDICINE.

Dr. K. N. Macdonald was fortunate enough, when acting as civil surgeon at Prome, British Burma, to obtain from a native practitioner, as a great favour, the loan of some palm-leaf manuscript purporting to be an exposition of the practice of medicine by the native physicians of the country. No clue to its age could be obtained, as the documents were undated, but in all probability it was as old as the Christian era. Owing to his being about to leave the district, Dr. Macdonald was unable to find time to transcribe the documents, but with the help of Mr. Mackertich, an able Oriental scholar and Government teacher, he translated them, and now publishes his work in the *Caledonian Medical Journal* for July 1910. After an historical survey of Burma and Buddhism from the earliest times, from which we gather that Prome was founded b.c. 444, he proceeds to deal with the medical teaching contained in the manuscript. It is impossible in the space at our disposal to do more than notice a few of the quaint theories with which Dr. Macdonald's translation abounds, or yet to describe in full the therapeutic methods and ample materia medica which he quotes. Still, a few words as to the physiology of the human body and the pathology of disease according to native ideas may be of interest. The circulation of the blood in the human body consists of two motions—a rising and an ebbing, each of which continues for six days alternately. On the seventh day the ascending and descending blood meet, during which time the least derangement will give rise to disease. The human organism is composed of four dâts or elements—namely: (1) The Pah-ta-wee or earth dât, consisting of the flesh, bones, fæces, etc. (2) The Tay-zawor fire dât—the heat, internal and external, of the animal body. (3) The Ah-baw or water dât—the blood, sweat, urine, marrow, and other fluids; and (4) the Wah-yaw or windy dât—the wind which is belched from the stomach. These four dâts are kept in motion by the Ah-Ka-tha or Heaven dât, and when any one of the component parts of either of the four is influenced by this fifth dât, that part of the body to which it belonged is put out of order. The day of the week upon which an illness starts has a marked influence in determining which part of the organism will be attacked. Thus on Sunday, because of the earth dât being in excess, the fire dât which attended the patient at his conception is destroyed, and the gall is in consequence touched. On Monday the appetite is lost through the water dât destroying the windy one. The gall is once more disordered on Tuesday by the fire dât being in excess. Wednesday sees the appetite destroyed by the triumph of the water dât, and on Thursday, by the disturbance of the heavenly and fire dâts, the gall is once again in excess. The destruction of the earth dât, together with an accumulation of foul food in the stomach, causes disturbance to the fire dât and so increases the heat in the system on Friday. Saturday sickness destroys the fire dât and disturbs that of water. No matter on which day it arises the disease will subside on the sixth day and disappear on the twelfth, but of course each day has its appropriate remedies which must be used to overcome the malign influence of the particular element which is in the ascendent. A long and protracted fever is called a Tha-nee-bat, and when it arises no medicine should be given the patient, for he will die in nine days if he refuses food and feels his throat very dry. If he become deaf he may live to the tenth day, but if his eyes are dim he will not live more than five days. Before giving medicine in any particular case the symptoms must be explained to the physician, for death or a prolonged illness may ensue if medicine be given when it is not required. A change of locality may be very beneficial to a patient, as such changes influence the elements and moderate those whose ascendency is causing the disorder. A sudden disease is caused by the five dâts counteracting each other, or by the inversion of the blood-stream, in which case the whole body becomes benumbed. Sweet toddy and jaggery, a kind of sugar, are then appropriate remedies. Affections of the eyes, ears, nose, tongue or any other part of the body may be caused by trouble and mental anxiety, or as the result of changes in the seasons, or of taking at an improper time food which disagrees with the constitution. Therefore, before resorting to any mode of treatment, the nature and cause of the disease should be first ascertained. Among general diseases are Lay-tha-lait or windly phlegm, Thway-tha-lait or bloody phlegm, Thai-gyee-tha-lait or galleous phlegm. Ngau or venomous influence on certain diseases is of three kinds. The first, or bloody Ngau, lasts three days, the second or windy variety four days, and the last, or galleous, five days. The patient either dies or is relieved on the third, seventh, or twelfth day after the first attack of any of these varieties of Ngau. Nghat Phyah or ague is caused by residence in cold and moist localities, and in hilly and mild regions. The patient may have secondary diseases, such as doolah (asthma), cough, and the spitting of phlegm. Bloody doolah is caused by the blood when it comes into contact with the urine, and windy doolah by the urine and putrid food, which is a secondary disease. Tounghoo-nah or Toonghoo distemper, an itchy disease and a species of leprosy caused by an excess of the windy bile, and peculiar to the district whence it takes its name, is a curable disease, but the Tha-min-yet or deer-licked disease, true leprosy, is incurable, and anyone who discovers a remedy for it may consider himself a fortunate man, for it has never yet been cured by anyone. Kyouk (a stone), or small-pox, is called the unavoidable disease from its being rooted in the body from the time of birth, and is occasioned by different causes. In addition to the five dâts, it may arise from excess of gall and from superfluity of blood. Seven varieties of the disease which are considered dangerous are mentioned. Diarrhœa is caused by the ascending or quick circulation, constipation by the descending weak or slow stream. An infant is liable to disease because of the heat absorbed from its mother's womb.

When ailing a sucking infant is not given medicine direct, but the mother is dosed, as the ailment can only be removed through the mother's milk. When a pregnant woman exposes herself to heat, cold, or wet, or is careless in diet, the child will be born diseased, and generally die on the sixth day. If it survive and the mother is still careless, the disease will become permanent or end in deformity. Children born prematurely are generally born sickly. No mention is made of midwifery proper, possibly, as the author suggests, because no man could be expected to interest himself particularly in diseases peculiar to such inferior beings as women are held to be in Burma. A woman's life is, however, divided into three periods—infancy and childhood from birth to the onset of catamenia, puberty while the menses last, and infirmity after the menopause. At the changes from one period into another they have the watery element in excess, but in the second period the fiery dât predominates. The windy dât leads to spotted bodies, the fiery dât to fever, weariness, loss of appetite, pallor, and dryness. The European doctor sees only really difficult midwifery cases in Burma, because it is only in such cases that he is called in. He has to save, if possible, the woman from dying in childbed, in which case she would be incapable of entering the state of Nigban or total annihilation—which is the Burmese heaven. Surgery would seem to be neglected as much as midwifery, but, as the author points out, only one book was seen, and in view of their extensive materia medica it is quite possible that the Burmese may have practised the setting of fractures, the healing of wounds, and the removing of necrosed bones.

